# "Propaganda" Films and Censorship

**Published:** 1921-01-08

**Authors:** 


THE PUBLIC WELL-BEING—(*continued*).

### "Propaganda" Films and Censorship.

More than once attention has been called in these columns to the danger to which children may be exposed, both mentally and physically, through the medium of the picture-houses—not because the films exhibited are in themselves improper, but because so many of them, particularly the long melodramatic serial, are calculated to produce an unhealthy excitement in a child of tender years. Now the cinema industry is greatly agitated about a problem germane to this matter, and one in which the public is vitally concerned. It has to do with the exhibition of what are known as propaganda films—more especially those pseudo-medical subjects which, in one form or another, pretend to explain these matters and warn the wary against moral pitfalls. Lately there have been produced, according to an apparently well-informed writer in the Press, a number of ingenious films (chiefly in America) which purport to explain sex dangers to the young and call public attention to the "social evil."

It is difficult to say whether these films are sincere or not—we do not pretend to judge—but they are at any rate, it is stated, "a hard nut for the censor to crack." Some bear his signature and some do not. The question is also complicated by the fact that the Board of Film Censors have asked the

THE PUBLIC WELL-BEING—(*continued*).

local authorities not to allow them to be shown in their several districts without the censor's certificate, while some of the local councils are against the films being shown, and others, partly through the pressure from their medical officers of health and from certain social organisations, are in favour of them. Furthermore, the Cinematograph Exhibitors' Association refuse to allow their members to show propaganda films in any city, and members have already been expelled for contravening the rule. It is a singular situation, and the conflicting views prevailing—particularly the attitude of the Exhibitors' Association—suggest that the whole question demands very close and impartial investigation. Without being "squeamish" on the subject it is obviously of great importance that some responsible authority should be able to give a definite assurance that films designed specifically to impart sex-education to the young, and therefore specially to invite the attendance of the young on high moral grounds, are really what they pretend to be; that there is nothing objectionable or in bad taste in the manner of their presentation; and that they are not used merely as a commercial exploitation through exciting prurient curiosity. Within legitimate and well-understood limits these "educative" films may serve a really valuable purpose: it is not difficult to see that under unscrupulous handling they may become a source of public danger.

The most practical proposal which has so far been made is that the Cinematograph Exhibitors' Association and the local councils should combine in an effort to see that sex-explanatory films are not shown for purposes of profit, and that they shall be shown under special conditions away from the ordinary cinema theatres. A further simple plan would be to lay down a fixed rule that no film of this kind should be passed for exhibition until it had survived the test of the local medical officer's personal scrutiny.

